# Using integrated correlative cryo-light and electron microscopy to directly observe syntaphilin-immobilized neuronal mitochondria *in situ*

**DOI:** 10.1007/s41048-017-0035-x

**Published:** 2017-03-21

**Authors:** Shengliu Wang, Shuoguo Li, Gang Ji, Xiaojun Huang, Fei Sun

**Affiliations:** 1 National Key Laboratory of Biomacromolecules, CAS Center for Excellence in Biomacromolecules, Institute of Biophysics, Chinese Academy of Sciences, Beijing, 100101 China; 2 Center for Biological Imaging, Institute of Biophysics, Chinese Academy of Sciences, Beijing, 100101 China; 3 University of Chinese Academy of Sciences, Beijing, China

**Keywords:** Correlative cryo-light and electron microscopy, iCorr, Mitochondria, Primary hippocampal neuron cell, Syntaphilin

## Abstract

Correlative cryo-fluorescence and cryo-electron microscopy (cryo-CLEM) system has been fast becoming a powerful technique with the advantage to allow the fluorescent labeling and direct visualization of the close-to-physiologic ultrastructure in cells at the same time, offering unique insights into the ultrastructure with specific cellular function. There have been various engineered ways to achieve cryo-CLEM including the commercial FEI iCorr system that integrates fluorescence microscope into the column of transmission electron microscope. In this study, we applied the approach of the cryo-CLEM-based iCorr to image the syntaphilin-immobilized neuronal mitochondria *in situ* to test the performance of the FEI iCorr system and determine its correlation accuracy. Our study revealed the various morphologies of syntaphilin-immobilized neuronal mitochondria that interact with microtubules and suggested that the cryo-CLEM procedure by the FEI iCorr system is suitable with a half micron-meter correlation accuracy to study the cellular organelles that have a discrete distribution and large size, *e.g.* mitochondrion, Golgi complex, lysosome, *etc*.

## INTRODUCTION

Fluorescence light microscopy (FM) offers large-scale, time-resolved, and dynamic visualization of positions of interest (POIs) in cells and provides a variety of information in cellular processes. The resolution of FM is restricted to ~200 nm due to diffraction limit (Abbe 1873), which was overcome by the recently developed super-resolution fluorescence microscopy techniques including photon-activated localization microscopy (PALM)/stochastic optical reconstruction microscopy (STORM) (Betzig *et al.* 2006; Hess *et al.* 2006; Rust *et al.* 2006), stimulated emission depletion fluorescence microscopy (STED) (Hell and Wichmann 1994; Klar *et al.* 2001), structured illumination microscopy (SIM) (Heintzmann and Cremer 1999; Gustafsson 2000; Li *et al.* 2015), *etc.* However, FM could only provide localization information of labeled molecules, but with a lack of structural context in the cells.

Electron microscopy (EM) has been applied into the study of cellular ultrastructures for many years and can provide detailed structural information of cells in nanometer-scale resolution. In addition, besides chemical fixation, cryo-vitrification technique has provided a good immobilization of cellular ultrastructures in their native state, which can be imaged by cryo-electron microscopy (cryo-EM) (Dubochet *et al.* 1988; Al-Amoudi *et al.* 2004). With the recent development of direct-detection devices, cryo-EM has been becoming one of the important tools in structural biology to resolve macromolecular structures in near-atomic resolution (Nogales and Scheres 2015). However, for cell biology study, EM technique is still restricted by a few limitations (Kukulski *et al.* 2011; Zhang 2013) due to (1) having a small field of view (FOV) and restricted specimen thickness; (2) being difficult to locate POI inside a cell; (3) being unable to capture dynamic state of events due to fixed specimen; and (4) being hard to distinguish specific molecules in a crowded cellular environment.

To combine the benefits of FM that provides localization information and EM that provides structural information, an emerging technique, termed correlative light and electron microscopy (CLEM), has been developed in the recent years (Mironov and Beznoussenko 2009; Hanein and Volkmann 2011). CLEM first localizes POI using FM and then transfers the localization information into EM and eventually generates two correlated images (fluorescence image and electron micrograph), within which the localizations of target molecules can be mapped onto their relevant structural contexts. To observe the biological structures in their native state, a particular technique, cryo-CLEM, obtained by correlating fluorescence cryo-microscopy (cryo-FM) with cryo-EM and imaging cryo-vitrified biological specimen, has received more and more attention in the recent years (Wolff *et al.* 2016). Several kinds of dedicated cryo-stages were developed to adapt standard fluorescence microscopes to perform cryo-FM (Sartori *et al.* 2007; Jun *et al.* 2011; Schorb *et al.* 2016). Fluorescent beads with enough electron density were used to correlate cryo-FM image into cryo-EM micrograph (Jun *et al.* 2011; Schorb *et al.* 2016). The cryo-vitrified specimen needs to be imaged by cryo-FM first and then transferred to the column of electron microscope for cryo-EM imaging.

Besides, to avoid specimen transfer that would cause severe ice contamination and specimen devitrification, an integrated cryo-CLEM workflow was developed, the so-called “iCorr” by the FEI Company, which integrates a fluorescence light microscope into the column of a transmission electron microscope (TEM). To use the iCorr system, the specimen loaded with a cryo-holder is rotated 90° to be imaged by fluorescence microscope (FM mode) and then rotated back to 0° for cryo-EM imaging (TEM mode). The iCorr system uses a LED illumination with the excitation wavelength ranging from 460 to 500 nm and with the peak at 470 nm. An optical filter in the iCorr system is used to pass through the emission light with the wavelength ranging from 510 to 560 nm. With a fixed objective lens having a numeric aperture (NA) of 0.5, the iCorr system can capture the fluorescence images at the green channel with the magnification of ×15 and the resolution of ~460 nm. Considering the early development stage of the iCorr system, there are a few cryo-CLEM applications of iCorr system in the literature. In the present work, we intend to test our prototype iCorr system integrated onto our Tecnai Spirit electron microscope by imaging cryo-vitrified rat hippocampal neuron cells.

Neuron cells are highly polarized and consist of three distinct structural and functional domains with unique morphologies: one with large and compact cell body that is called soma; one with thin and long axon; and one with numerous thick dendrites with branches. Axon and dendrites are also called neuronal processes. In neuron cells, mitochondria, as essential organelles for energy production, intracellular calcium homeostasis maintenance, and steroid and lipid synthesis (Nicholls and Budd 2000; Boldogh and Pon 2007), are transported between processes and soma according to energy and metabolic requirements at different regions (Hollenbeck and Saxton 2005). The transport of neuronal mitochondria has been extensively studied by using time-lapse fluorescence microscopy (Misgeld *et al.* 2007; Kang *et al.* 2008). Mitochondria can move a long distance without stopping or frequently changing direction, pausing or persistent dwelling (Hollenbeck and Saxton 2005; Misgeld *et al.* 2007; Kang *et al.* 2008). The movements of mitochondria are majorly along microtubules (Nangaku *et al.* 1994; Goldstein and Yang 2000). In mature neuron’s axons, only approximately one-third of mitochondria are mobile while the remaining being stationary (Kang *et al.* 2008).

The molecular mechanism of mitochondrial station in neuron cell has been studied for many years. Syntaphilin (SNPH) has been found as a neuron-specific protein that locates on the mitochondrial outer membrane and binds to microtubule and would be a stationary factor for mitochondrial immobilization. Knock out SNPH gene resulted in a dramatic increase of mobile axonal mitochondria; however, overexpressing SNPH protein could immobilize almost all of the axonal mitochondria (Kang *et al.* 2008). Although SNPH has been revealed as a receptor for immobilizing mitochondria in axons, little is known about its molecular mechanism.

In the present study, we labeled SNPH with the fluorescent tag Dendra2 and mitochondria with the fluorescent marker TagRFP-mito in the rat hippocampal neuron cells and utilized the cryo-CLEM approach with iCorr to image the syntaphilin-immobilized neuronal mitochondria *in situ*.

## RESULTS

### Culturing hippocampal neuron cells on grids and cryo-vitrification

**Fig. 1:**
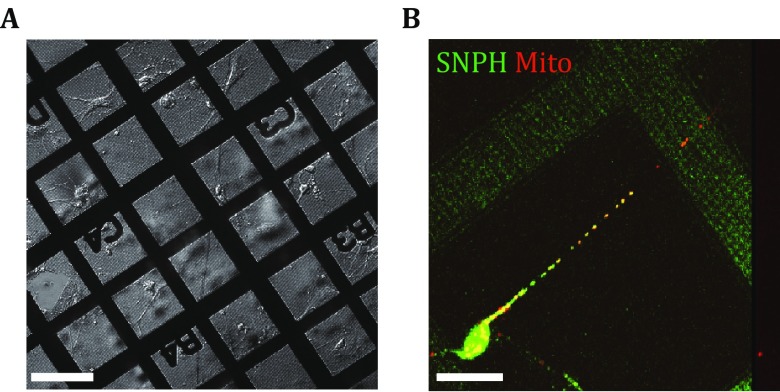
Rat hippocampal neuron cells grown on EM grids. **A** Differential interference contrast light microscope image of rat primary hippocampus neurons cultured on EM grids for 9 d. Scale bar, 100 μm. **B** Fluorescent visualization of neurons cultured on EM grids that were cotransfected at DIV of 6 with Dendra2-SNPH (*green*) and TagRFP-mito (*red*). The colocalized regions are shown in *yellow*. Scale bar, 20 μm

Rat hippocampal neuron cells can grow on the carbon film-coated gold EM grid with healthy morphology (Fig. 1A). After transfection with the plasmids of Dendra2-SNPH and TagRFP-mito, the cotransfected cells showed that all axonal mitochondria became immobilized as previously reported (Kang *et al.* 2008), and the fluorescence signals from SNPH and mitochondria were significantly colocalized (Fig. 1B). With the successful neuron cell culturing and transfection, we were ready for the further cryo-CLEM experiments.

**Fig. 2:**
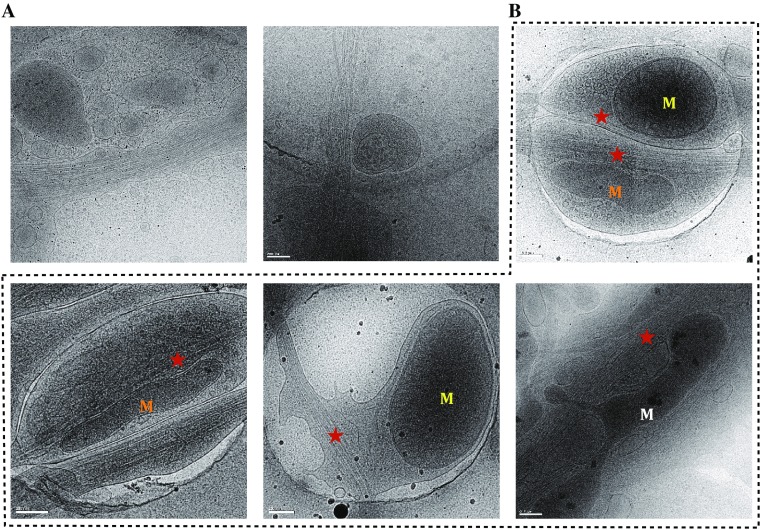
Cryo-electron micrographs of vitrified rat hippocampal neuron cells (nontransfected) at process regions. **A** Broken cells caused by double-sided blotting method in plunge freezing. Scale bar, 200 nm. **B** Intact cells vitrified by single-side blotting method in plunge freezing. The mitochondria with round, elongated, and branched shapes are labeled in *yellow*, *orange,* and *white*, respectively. Microtubules are indicated with a *red star*. Scale bar, 200 nm

Before applying cryo-CLEM to observe the SNPH-immobilized mitochondria, we first utilized those nontransfected neuron cells to explore an appropriate freezing method to vitrify the grids where the cells grow on. Plunge-freezing method has been successfully applied to cryo-preserve the neuron cells cultured on EM grids (Lucic *et al.* 2007). In the present study, we aimed to utilize FEI Vitrobot device to perform plunge freezing. However, we found that the double-side blotting method by FEI Vitrobot did not work well for freezing the fragile neuron cells that were easily broken down due to the blot force from the double sides (Fig. 2A). To overcome this problem, we removed one blot pad from one side, and let the filter paper from another side to blot the backside of the grid where no neuron cells were growing on. With this single-side blotting approach, we were able to vitrify the neurons in their native states (Fig. 2B). The neuronal processes, particularly the axons, could be embedded in thin (200–500 nm) layer of ice and readily be imaged by cryo-EM, and the double layer of mitochondria membrane and the architecture of microtubule as well as trafficking vesicles were all clearly visible (Fig. 2B).

We noticed that mitochondria in the neuronal processes have variable morphologies including tubular, branched, and round. Moreover, we also observed the interaction between some mitochondria and microtubule and some not (Fig. 2B), which are actually relevant to different physiological states of mitochondrion. Whether SNPH overexpression could enhance such interaction and increase the distribution of the microtubule interacted mitochondrion needs to be further investigated by the subsequent cryo-CLEM experiments.

### Cryo-CLEM of the SNPH transfected hippocampal neuron cells

Clonable fluorescent protein has been reported to have an unanticipated advantage in reducing the rate of fluorescence photo bleaching in cryogenic temperature and very suitable for cryo-CLEM experiments (Schwartz *et al.* 2007). In the present work, the nonactivated Dendra2, a GFP variant, which possesses excitation–emission maxima at 490 and 507 nm similar to EGFP and other green fluorescent proteins (Gurskaya *et al.* 2006), was selected to label the SNPH protein. Considering the excitation wavelength range (460–500 nm) and emission wavelength range (510–560 nm) of the iCorr system, Dendra2 is suitable (but not optimized) for cryo-CLEM work using the iCorr.

**Fig. 3:**
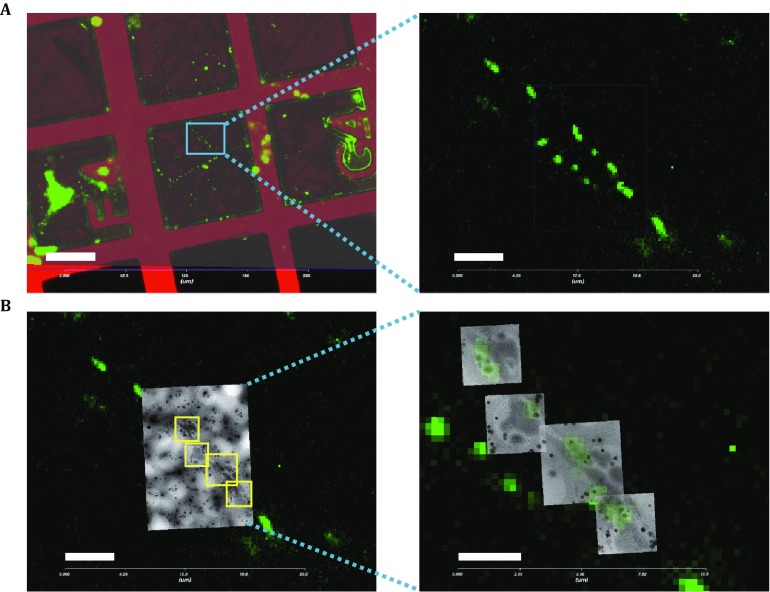
Applying the cryo-CLEM procedure to the vitrified SNPH-transfected rat hippocampal neuron cells. **A** The reflection image (*red*) is merged with the corresponding fluorescence image (*green*). Scale bar, 50 μm. Region of interest (ROI) is selected by *blue box* and magnified at right. Scale bar, 5 μm. **B** Medium magnification transmission electron microscopy (TEM) image is first taken in the center of ROI, which is automatically correlated to the FM image with slight manual modification. Then, this correlated image is further used to select areas (indicated by *yellow box*) for high-magnification TEM imaging. Scale bar, 5 μm. The final merged and correlated TEM and FM images with high magnification are shown at right. Scale bar, 2.5 μm

**Fig. 4:**
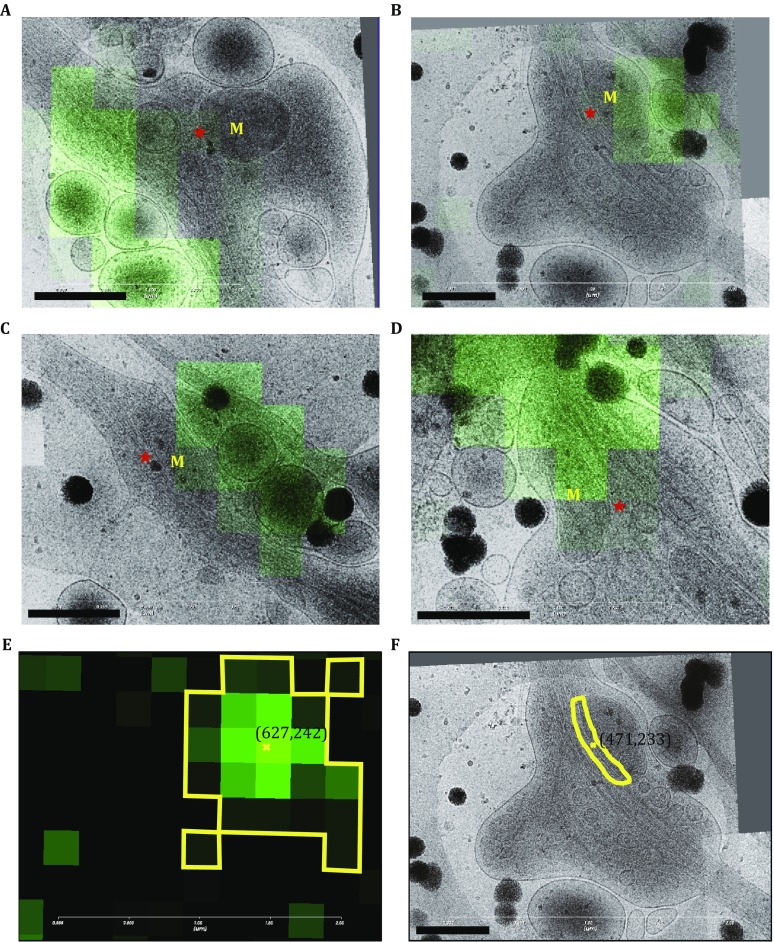
Correlation between cryo-FM and cryo-EM images. **A–D** Aligned and overlaid cryo-FM and cryo-EM images located at the regions of interest in Fig. 3B from top to bottom. The mitochondria are labeled in *yellow,* and microtubules are indicated with *red stars*. Scale bar, 0.5 μm. (**E**, **F**) Correlation accuracy between cryo-FM and cryoEM images is measured on the basis of the center of the signals. In cryo-FM image (**E**), the contours in *yellow* represent the profile of the fluorescent signal. In addition, in cryo-EM image (**F**), the contours in *yellow* represent the interacting region between mitochondria and microtubule, where SNPH are localized. The *yellow* crosses represent the centers of the contours with the corresponding coordinates in pixel. Scale bar, 500 nm

The entire process of cryo-CLEM is shown in Fig. 3. First, the reflective and fluorescent images with a large field of view were acquired and merged (Fig. 3A, left). Then, the region of interest was selected and magnified (Fig. 3A, right) for the subsequent cryo-EM imaging. A cryo-EM image in a medium magnification with a field of view (~10 × 10 μm^2^) was acquired and automatically correlated with the cryo-FM image (Fig. 3B, left). With the benefit of holey carbon film, we manually slightly optimized the translation and rotation alignments between cryo-FM and low-magnification cryo-EM images. Thereafter, the subsequent cryo-EM images at a high magnification was acquired and aligned to the magnified cryo-FM images (Fig. 3B, right). The correlated images showed that the Dendra2-SNPH fluorescent signals had located along the neuron cell process. For each discrete fluorescence signal, one single mitochondria organelle was found nearby with elongated or round morphologies (Fig. 4A–D). We also found that the elongated mitochondria closely interact with the microtubules along its long axis (Fig. 4B–D), while the mitochondria in the round shape loosely interact with the microtubule via a small region (Fig. 4A).

Since SNPH proteins are colocalized with the discretely distributed mitochondria in the axonal process (Fig. 1B), we were confident to identify the fluorescent signals of SNPH-Dendra2 in the cryo-FM images, and the nearby-located mitochondria in the cryo-EM images were actually correlated (Fig. 4A–D). Upon this assumption, the correlation accuracy of this experiment could be estimated (see next section).

From the cryo-CLEM images of SNPH-transfected hippocampal neuron cells, it would be surmised that the SNPH-immobilized mitochondria in the neuronal axon vary in the morphologies from elongated to round, which is similar to the previous observation of the glutamine-induced mitochondrial immobilization (Rintoul *et al.* 2003).

### Correlation accuracy estimation

The correlation accuracy between cryo-FM and cryo-EM depends on many factors including the resolution of cryo-FM itself, the distortion variations from different imaging systems, and the alignment accuracies of different sources of images. Here, we used the final correlated images to determine the overall correlation accuracy of our iCorr system.

The fluorescence signal of each correlated cryo-FM image (Fig. 4A–D) represents many SNPH molecules located on the outer membrane of mitochondria and thus the center of each fluorescence spot represents the central position of a cluster of SNPH molecules from one mitochondrion (Fig. 4E). Since SNPH mediates the direct interaction between mitochondria and microtubule and locates at their direct contacts, the center of the contact interface between mitochondria and microtubule also represents the central position of SNPH molecules from one mitochondrion (Fig. 4F). Comparing the shift between these two centers computed from two different sources of images would give an estimation of the correlation accuracy in this cryo-CLEM experiment. As shown in Table 1, from the four correlated images (Fig. 4A–D), the mean shift between these two centers was 488.5 ± 121.8 nm, which is close to the resolution (~460 nm) of the FM in the iCorr system, suggesting that the limitation factor of the correlation accuracy in the iCorr system is the resolution of its FM module.

**Table 1:** Signal shifts between the correlated cryo-FM and cryo-EM images that were captured in the iCorr system^a^

	Center of MM^b^ interaction site in cryo-EM image		Center of SNPH^c^ fluorescence in cryo-FM image		Pixel size (nm/pixel)	*S* ^d^ (nm)
	*X′* (pixel)	*Y′* (pixel)	*X* (pixel)	*Y* (pixel)		
1	453	259	253	463	2.2	629.3
2	471	234	627	242	2.8	436.8
3	352	300	594	337	2.2	539.0
4	461	355	381	178	1.8	348.9
					*S* ^d^ (nm)	488.5
					*Sd* ^d^ (nm)	121.8

^a^ FEI’s iCorr technology that consists of a fluorescence light microscope module, which is integrated with FEI’s Tecnai transmission electron microscope, and a software for automatically correlating FM and EM images. Location information 1–4 are from the four correlated images (Fig. 4A–D) respectively

^b^ Mitochondria and microtubule

^c^ Syntaphilin, which has a role for maintaining a large number of axonal mitochondria in a stationary state on microtubule, is labeled with Dendra2 fluorescent protein

^d^ The shift between two centers of signals (see Materials and Methods)

## CONCLUSION

In the present work, we tested the cryo-CLEM procedure based on the FEI iCorr system and applied this technique to the cryo-vitrified rat hippocampal neuron cells that were transfected with Dendra2-SNPH. We developed a successful protocol using FEI Vitrobot to freeze the fragile neuron cells grown on grids and preserve them in their native state. We directly visualized the SNPH-immobilized neuronal mitochondria *in situ* and successfully captured the varied morphologies of the SNPH-immobilized mitochondria as well as their interactions with microtubules. The estimated accuracy of the correlation between cryo-FM and cryo-EM was 488.5 ± 121.8 nm, suggesting that the current cryo-CLEM procedure by the FEI iCorr system would be suitable for cryo-CLEM study of cellular organelles like mitochondrion, Golgi complex, lysosome, and so on, which have a discrete distribution and large size.

## MATERIALS AND METHODS

### EM grids preparation

Gold EM-grids with Alpha-Numeric Finder (G200F1-G3, Gilder Grids Ltd.) were coated with holey carbon (2-μm round hole spaced by 2 μm), which was manufactured in house. All the grids were sterilized by ultraviolet light overnight before transferred into culture dishes that were coated with Matrigel matrix (Product #354248, Coring life science). The grids were put on the surface of Matrigel matrix with the carbon side on the top.

### Neuron cell cultures and transfection

Primary hippocampal neuron cells were dissected from postnatal day 0–1 Spraque–Dawley rats in accordance with the procedures in Kang’s lab, the Institute for Nutritional Sciences, SIBS, CAS. Briefly, hippocampal neurons were dissected in cold HBSS buffer (H2387, Sigma), digested in a DNase/trypsin solution (0.5 mg/mL DNase, 5 mg/mL trypsin, 25 mmol/L HEPES, 137 mmol/L NaCl, 5 mmol/L KCl, 7 mmol/L Na_2_HPO_4_, pH 7.2) for 5 min at 37 °C, and then dissociated into separated single cells in a DNase solution (0.5 mg/mL DNase, 25 mmol/L HEPES, 137 mmol/L NaCl, 5 mmol/L KCl, 7 mmol/L Na_2_HPO_4_, pH 7.2). After washing with HBSS and 10% FBS, neurons were plated on a Matrigel matrix coated culture dish with EM grids on the top. For cryo-CLEM application, low-density neuron cells were kept at 37 °C in 5% CO_2_. At days *in vitro* (DIV) of 6–9, the cells were transfected with the plasmids of Dendra2–SNPH and TagRFP-mito using the calcium phosphate method (Jiang and Chen 2006). After transfection, the cells were cultured further for additional 2–3 days before inspection using a laser scanning confocal fluorescence microscope (FV1000, Olympus), and the transfected cells were identified according to the fluorescence signals of Dendra2 and TagRFP. The regions of processes where SNPH are overexpressed were selected and marked using the nearest alpha-numeric finder in the grid.

### Cryo-vitrification of hippocampal cells

The EM grids with neuron cells grown on were cryo-vitrified using FEI Vitrobot (Mark IV). To achieve single-side blotting that is important to keep the integrity of the fragile neuron cells, one blot pad of Vitrobot was removed before the vitrification process. The EM grids with the grown neuron cells were carefully picked up from the dishes using a Vitrobot tweezer (FEI), and washed once in a dish with warm HBSS buffer (H2387, Sigma). After applying additional 3 μL HBSS buffer onto the side where neurons have grown, the tweezer with the grid was mounted onto Vitrobot by allowing the side of neurons to be facing the side of the removed blot pad. As a result, the excess liquid was blotted by the filter paper from the backside of the grid, and there is no direct contact between the filter paper and the cells. The following parameters were set up during blotting: blot force 8, blot time 8 s, temperature 25 °C, and humidity 100%. After blotting, the grid was rapidly frozen in liquid ethane that was precooled by liquid nitrogen and transferred to liquid nitrogen for storage.

### Cryo-CLEM of the vitrified cells

The vitrified grid was mounted into a cryo-holder (Model 626, Gatan) that was precooled in liquid nitrogen. Then the cryo-holder was loaded into the column of the transmission electron microscopy Tecnai Spirit that is supplied with the FEI iCorr module. The regions with the marked finders, which were selected prior to vitrification, were searched and centered in the TEM low-magnification mode. Then, the subsequent cryo-CLEM operations were performed in the software of the iCorr system.

The FM mode was first selected, and the stage was tilted to the angle of 90°. Since most of the liquid nitrogen stored in the cryo-holder Dewar spilled out when tilting at 90°, the image acquisition in FM model should be finished within 30 min to prevent warming up the specimen. Both reflection and fluorescence images were recorded by adjusting the *Z*-focus value and optimizing the light illumination intensity. Then, the stage was tilted back to 0° for EM model operation. Positions of interest (POIs) with fluorescent signals were selected, and the cryo-EM images with medium magnification and FOV of ~10 × 10 μm^2^ were acquired. The cryo-EM images were automatically correlated to the cryo-FM images by the software using the precalibrated parameters for translation, rotation, and scaling. This initial correlation might not be sufficiently correct due to the mechanical error of the stage and the drift of the specimen. To optimize the correlation between the cryo-EM and cryo-FM images, repositioning manually according to the features of carbon holes in both reflection and cryo-EM images was performed. After correlation optimization, the final cryo-EM micrographs targeted at the higher magnification were acquired by clicking the POIs in the correlated FM–EM image using the software of the iCorr system. All the procedure for cryo-EM imaging were controlled in a low-dose condition.

### Quantification of the correlation accuracy

The correlated cryo-FM and cryo-EM images were separately saved in a PNG format. Then, these images were uploaded into Image J software (Schneider *et al.* 2012). For the cryo-FM image, a polygon selection tool was used to contour the profile of the entire fluorescence spot, and then the center of the fluorescence signal was calculated using the tool of “measuring the center of mass”. The coordinates of the center were denoted by (*X*_*i*_, *Y*_*i*_) in pixels. For the cryo-EM image, a freehand selection tool was used to contour the interacting site between mitochondria and microtubule, which was the position where SNPH proteins are localized. The tool of “measuring the center of mass” was also used to calculate the center of the SNPH-localized region with the coordinates denoted by (*X*_*i*_^′^, *Y*_*i*_^′^) in pixels. Thus, after pixel size correction, the shift *S*_*i*_ of the correlated signals between cryo-FM and cryo-EM images were calculated as follows:

$$S_{i} = \sqrt {\left( {X_{i} - X_{i}^{'} } \right)^{2} + \left( {Y_{i} - Y_{i}^{'} } \right)^{2} }$$

The mean shift *S* and its standard deviation *Sd* were calculated as follows:

$$\bar{S} = \frac{1}{n}\sum\limits_{i = 1}^{n} {S_{i} }$$

$$Sd = \sqrt {\frac{{\sum {\left( {S - \bar{S}} \right)^{2} } }}{n - 1}}$$
